# Pharmacogenomic and *in silico* identification of isoform-selective AKT inhibitors from *Pithecellobium dulce* for precision cancer therapy

**DOI:** 10.3389/fphar.2025.1744408

**Published:** 2026-02-03

**Authors:** Gnanaprakash Jeyaraj, Bing Yang, Kuppusamy Sathishkumar, Santosh Chokkakula, Bader O. Almutairi, Weimin Xie

**Affiliations:** 1 Saveetha Medical College and Hospital, Saveetha Institute of Medical and Technical Sciences, Chennai, Tamil Nadu, India; 2 Department of Public Health, International School, Krirk University, Bangkok, Thailand; 3 Department of Biotechnology, Rathinam College of Arts and Science, Coimbatore, Tamil Nadu, India; 4 Department of Microbiology, Chungbuk National University College of Medicine and Medical Research Institute, Cheongju, Chungbuk, Republic of Korea; 5 Department of Zoology, College of Science, King Saud University, Riyadh, Saudi Arabia; 6 Department of Gynecology, Affiliated Hengyang Hospital of Hunan Normal University & Hengyang Central Hospital, Hengyang, Hunan, China

**Keywords:** AKT1/AKT2 inhibition, isoform selectivity, molecular dynamics, natural product drug discovery, Pithecellobium dulce

## Abstract

**Objective:**

AKT1 and AKT2 are central but functionally distinct kinases within the PI3K–AKT–mTOR pathway, and isoform‐specific genomic alterations in these proteins have important implications for cancer prognosis and therapeutic responsiveness. This study aimed to integrate cancer pharmacogenomics with structure‐based modeling to identify natural compounds capable of selectively targeting AKT1 or AKT2.

**Methods:**

Public cancer genomics datasets from TCGA and the Kaplan–Meier Plotter were analyzed to characterize mutation patterns, copy number alterations, and survival associations of AKT1 and AKT2 across malignancies. Based on isoform-specific differences, twenty phytochemicals from Pithecellobium dulce were docked against the allosteric binding sites of AKT1 (PDB: 3QKL) and AKT2 (PDB: 2JDO). Lead compounds were evaluated using ADME prediction and density functional theory to assess pharmacokinetic suitability and electronic stability. The dynamic behavior of ligand–protein complexes was examined through 200‐ns molecular dynamics simulations using the Desmond–Schrödinger platform, and binding free energies were estimated via MM‐GBSA analysis. Regulatory interactions involving AKT‐associated non‐coding RNAs were also examined to support pharmacogenomic relevance.

**Results:**

Genomic analysis revealed that AKT1 alterations were dominated by activating missense mutations, particularly the E17K hotspot, whereas AKT2 showed frequent gene amplifications that were significantly associated with poor overall survival. Docking studies demonstrated clear isoform selectivity among P. dulce phytochemicals: oleanolic acid and pitheduloside I preferentially bound AKT1, while rutin and naringin exhibited stronger affinity toward AKT2. Oleanolic acid and rutin displayed binding energies comparable to established allosteric AKT inhibitors. ADME and DFT analyses supported favorable drug‐likeness and molecular stability of the lead compounds. Molecular dynamics simulations confirmed stable complex formation with persistent hydrogen bonding, and MM‐GBSA calculations indicated superior binding energetics for oleanolic acid–AKT1 and rutin–AKT2 complexes relative to controls. In parallel, analysis of miR-149‐5p and lncRNA HOTAIR highlighted post‐transcriptional regulatory mechanisms influencing AKT isoform activity.

**Conclusion:**

This study demonstrates that integrating pharmacogenomic profiling with multiscale molecular simulations can reveal isoform‐specific vulnerabilities within the AKT signaling axis. Phytochemicals derived from Pithecellobium dulce, particularly oleanolic acid and rutin, emerge as promising selective modulators of AKT1 and AKT2, respectively. These findings provide a mechanistic and structural foundation for the development of isoform‐guided AKT‐targeted therapies and support further experimental validation toward precision oncology applications.

## 1 Introduction

The PI3K–AKT–mTOR signaling pathway is one of the most commonly dysregulated pathways in human cancers, controlling essential cellular processes such as survival, growth, metabolism, and migration. Aberrant activation of this pathway contributes to tumorigenesis, metastasis, and resistance to therapies (Fakhri et al., 2021; He et al., 2021). The AKT family consists of three isoforms: AKT1, AKT2, and AKT3, which are highly homologous serine/threonine kinases that execute distinct and sometimes opposing biological roles (Adon et al., 2025). AKT1 is primarily involved in regulating cell survival and proliferation, whereas AKT2 is more critical for metabolic control and insulin signaling (Tewari et al., 2022).

From a pharmacogenomic perspective, isoform-specific mutations and copy-number alterations in AKT1 and AKT2 stratify patient subgroups with differential prognosis and therapeutic sensitivity. The activating E17K mutation in AKT1, frequently detected in breast, colon, and lung cancers, causes constitutive membrane localization and downstream signaling activation (Gao et al., 2021; Adon et al., 2025). In contrast, AKT2 is often amplified in ovarian, pancreatic, and endometrial malignancies, correlating with aggressive phenotypes and poor clinical outcome (Fakhri et al., 2021). Such genomic heterogeneity forms the basis for pharmacogenomic classification in targeted therapy, where identifying isoform-specific vulnerabilities could optimize drug response and minimize adverse effects.

Despite their structural similarity, the functional divergence of AKT isoforms has complicated the development of selective inhibitors. Current pan-AKT inhibitors, including MK-2206 and Ipatasertib, inhibit multiple isoforms simultaneously, leading to dose-limiting toxicities such as hyperglycemia and skin rash (Lin et al., 2013; Saura et al., 2017; Harun et al., 2023). Moreover, adaptive resistance frequently emerges through secondary mutations in AKT or compensatory activation of parallel signaling routes (Fakhri et al., 2021). Hence, pharmacogenomic insights into AKT isoform dysregulation provide a rationale for developing selective AKT1 or AKT2 inhibitors capable of tailoring treatment to the patient’s mutational background.

Post-transcriptional regulation further modulates this pharmacogenomic axis. Non-coding RNAs (ncRNAs) including microRNAs (miRNAs) and long non-coding RNAs (lncRNAs) have been shown to fine-tune AKT signaling. For instance, miR-149-5p and miR-29b suppress AKT1 translation, whereas lncRNAs such as HOTAIR and MALAT1 enhance AKT2 activation by sponging inhibitory miRNAs or facilitating upstream PI3K signaling. Dysregulation of these ncRNAs contributes to oncogenic AKT signaling and therapeutic resistance, positioning ncRNA–AKT interactions as an additional layer of pharmacogenomic control in cancer [9–11].

Natural products represent a potential source of isoform-selective modulators, although their predicted activities require experimental confirmation. Owing to their structural diversity and multitarget potential, phytochemicals can modulate oncogenic pathways while reducing off-target toxicity. Several compounds including quercetin, epigallocatechin gallate (EGCG), and rutin have demonstrated the ability to inhibit AKT phosphorylation and promote apoptosis (Naeem et al., 2022; Tewari et al., 2022). These compounds can also modulate upstream and downstream signaling pathways, enhancing their therapeutic potential by addressing multiple facets of tumorigenesis and resistance.

Among natural sources, *Pithecellobium dulce* (Manila tamarind) is rich in phenolics such as rutin, catechins, and gallic acid derivatives that exhibit potent anticancer activities by modulating PI3K/AKT signaling (Tewari et al., 2022). Extracts of *P. dulce* have been reported to downregulate KRAS and suppress MAPK/ERK and PI3K/AKT cascades, leading to apoptosis in colon carcinoma cells (Vargas-Madriz et al., 2025). *In vivo* studies also support the therapeutic potential of *P. dulce*. Dhanisha et al. (Dhanisha et al., 2022) reported that a fruit extract of *P. dulce* (FPD) inhibited metastasis in a melanoma mouse model, reducing the number of pulmonary tumor nodules and blocking pro-inflammatory signaling pathways.

Building on these pharmacogenomic and ncRNA insights, the present study investigates phytochemicals from *P. dulce* as potential isoform-selective inhibitors of AKT1 and AKT2. Using integrated computational strategies TCGA-based genomic analysis, molecular docking, ADME profiling, density functional theory (DFT), and long-timescale molecular dynamics simulations we delineate the differential binding features of these phytochemicals relative to clinically evaluated AKT inhibitors (MK-2206 and Ipatasertib). The findings aim to define structure-guided templates for selective AKT inhibition and provide a pharmacogenomic rationale for precision targeting of AKT-driven cancers.

## 2 Materials and methods

### 2.1 Genomic data analysis

Differential expression analysis of AKT1 and AKT2 was conducted using two publicly available platforms. GEPIA2 (http://gepia2.cancer-pku.cn/), this tool integrates RNA-seq data from 9,736 tumor samples and 8,587 normal samples from TCGA and GTEx projects [1]. The “Expression DIY” module was used to compare AKT1 and AKT2 expression between tumor and normal tissues across multiple cancer types. Default parameters were applied. TNMplot (https://tnmplot.com/analysis/), this platform compares gene expression across normal, tumor, and metastatic tissues using integrated microarray and RNA-seq datasets [2]. The “Normal–Tumor” comparison module was used to analyze log2-transformed TPM values for AKT1 and AKT2. Boxplots were generated to visualize expression patterns. Both tools consistently highlighted cancer-specific dysregulation of AKT1 and AKT2. Figures were generated directly from the respective platforms.

### 2.2 Mutation and prognostic analysis

To complement expression profiling, the mutation spectrum and clinical relevance of *AKT1* and *AKT2* were examined using publicly available cancer genomics platforms. The cBioPortal database (https://www.cbioportal.org) was queried across *The Cancer Genome Atlas (TCGA) Pan-Cancer Atlas* studies to identify the frequency and type of genetic alterations, including single-nucleotide substitutions, insertions/deletions, and copy-number amplifications. Mutation distributions were visualized using lollipop plots to highlight recurrent hotspots such as *AKT1 E17K* and *AKT2 R170W/Q* within the Pleckstrin Homology (PH) domains, and their frequencies across cancer types were summarized using the *Cancer Types Summary* module. Copy-number alterations (deep deletion, shallow deletion, diploid, gain, and amplification) and mRNA-expression correlations were analyzed through the *Plots* module using RSEM-normalized RNA-Seq data (log_2_ values) to determine dosage-dependent expression changes. Pathway-level alterations were examined through the *Pathways* module to map *AKT1* and *AKT2* within the PI3K–AKT–mTOR signaling cascade and to quantify co-alterations among genes such as *PIK3CA*, *PTEN*, *MTOR*, *RPTOR*, and *RICTOR*. Co-occurrence and mutual-exclusivity analyses were performed with the *Comparison/Survival* module to identify significantly co-altered genes, which were visualized through enrichment scatter and volcano plots.

The potential prognostic value of *AKT1* and *AKT2* expression was assessed using the Kaplan–Meier Plotter (http://kmplot.com/analysis), which integrates gene-expression data with clinical outcomes. Overall- and relapse-free-survival curves were generated using the auto-select cutoff, and hazard ratios (HR) with 95% confidence intervals were obtained using the platform’s in-built Cox regression analysis. Statistical significance was determined by the log-rank test (*P* < 0.05). Cancer-type-specific alteration frequencies were ranked across TCGA cohorts, and all figures including mutation lollipops, copy-number–expression plots, survival curves, and pathway diagrams were exported directly from cBioPortal in high-resolution format and standardized for publication.

### 2.3 Phytochemical and protein preparation

Twenty phytochemicals previously identified from *Pithecellobium dulce* were selected based on the comprehensive phytochemical profiling reported by Murugesan et al. (2019). Chemical structures were obtained from the PubChem database and subjected to energy minimization using Open Babel in PyRx 0.8, employing the Universal Force Field (UFF) and the conjugate gradient algorithm. Minimized ligands were then converted to PDBQT format for molecular docking. Crystal structures of AKT1 (PDB ID: 3QKL) and AKT2 (PDB ID: 2JDO) were retrieved from the RCSB Protein Data Bank. Water molecules were removed, hydrogen atoms added, and Kollman charges assigned using Chimera, followed by conversion to PDBQT format after optimization for docking analysis. Known allosteric inhibitors MK-2206 for AKT1 and Ipatasertib for AKT2 were used as reference control compounds to validate docking accuracy.

### 2.4 Structure-based virtual screening and molecular docking

Structure-based virtual screening was performed with AutoDock Vina implemented in PyRx 0.8. Docking grids were centered at the ATP-binding pockets of both AKT1 and AKT2 to encompass the active sites. The exhaustiveness parameter was set to 8. For each compound, the docking pose exhibiting the lowest binding energy was selected. Docking accuracy was validated by redocking native ligands. Protein-ligand complexes were visualized with BIOVIA Discovery Studio and PyMOL to identify hydrogen bonds and hydrophobic interactions, and to analyze binding modes.

To assess the reliability of the docking workflow, the co-crystallized ligands from the AKT1 and AKT2 crystal structures were extracted and redocked into their native binding pockets using the same Vina parameters. The reproduced poses showed close alignment with the crystallographic orientations, with RMSD values within the accepted range for accurate redocking (0.86 Å for AKT1 and 0.72 Å for AKT2). The preservation of key hydrogen-bonding and hydrophobic interactions confirmed that the docking protocol generated biologically meaningful binding modes.

### 2.5 ADME prediction and drug-likeness evaluation

The top docked ligands were evaluated for pharmacokinetic and drug-likeness profiles using SwissADME and the SCFBio Lipinski rule portal. Properties such as molecular mass, cLogP, hydrogen bond donors and acceptors, and molar refractivity were assessed. Compounds that satisfied at least four out of five Lipinski criteria were considered as having adequate oral bioavailability potential.

### 2.6 Density functional theory (DFT) calculations

DFT single-point energy calculations were conducted for the top-scoring phytochemicals using the RB3LYP functional and the 6-31G (d,p) basis set. Total energies, dipole moments, and the presence or absence of imaginary frequencies were determined to evaluate molecular stability and polarity. All calculations were performed on energy-minimized ligand structures to provide quantum descriptors relevant to drug binding and reactivity.

### 2.7 Molecular dynamics simulations

Dynamic stability of selected protein-ligand complexes was explored using the Desmond (v5.2) Schrodinger suite (version 2019-1) employing the OPLS-2005 force field. The selected ligand–protein complexes *AKT1*–oleanolic acid, *AKT1*–MK-2206, *AKT2*–rutin, and *AKT2*–Ipatasertib were solvated in an orthorhombic TIP3P water box with a 10 Å buffer distance from the protein surface and neutralized with appropriate numbers of sodium and chloride ions. Systems were equilibrated under NPT conditions at 310 K and 1.01325 bar. A 200-nanosecond MD production trajectory was recorded and analyzed to compute root mean square deviation (RMSD), root mean square fluctuation (RMSF), radius of gyration, and hydrogen bond dynamics. Additionally, protein–ligand contact maps and interaction histograms were extracted to evaluate the persistence of key interactions throughout the simulation, providing comparative insights into the stability and binding dynamics of the phytochemicals relative to the control compounds. The 200-ns production time was selected to ensure sufficient equilibration and to capture stable interaction patterns of the AKT–ligand complexes, consistent with standard practice in kinase-focused MD studies. Analysis of RMSD and RMSF profiles showed early stabilization and sustained convergence across trajectories, confirming that the simulation length was adequate for reliable comparative stability assessment.

### 2.8 MM-GBSA binding free energy calculations

Binding free energies were estimated using the Prime MM-GBSA module in Schrödinger (2019-1). The VSGB 2.1 solvation model and OPLS-2005 force field were applied. For docking-derived complexes, MM-GBSA was performed on minimized protein–ligand structures. For MD-derived complexes, the thermal_mmgbsa.py script was employed to extract every 10th frame from the last 50 ns of the trajectory, averaging ΔG_bind across 50 frames. Binding free energy components, including van der Waals, Coulombic, lipophilic, hydrogen bond, and solvation contributions, were analyzed.

### 2.9 Data analysis and visualization

Analytical and visualization steps including 2D interaction diagrams, RMSD/RMSF plots, contact maps, and histograms were carried out using Discovery Studio Visualizer, yMOL, and appropriate plotting tools.

## 3 Results

### 3.1 Genomic expression profiling of AKT isoforms

**FIGURE 1:**
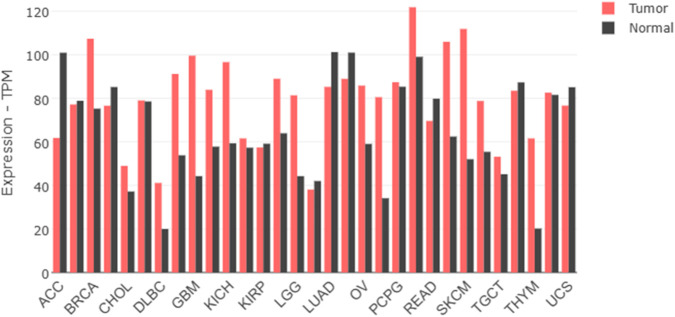
Gene expression of protein kinase B isoform 1 (AKT1) across multiple cancer types (GEPIA2). Boxplot showing AKT1 expression in tumor (right) and normal (left) tissues using TPM (Transcripts Per Million) values retrieved from the Gene Expression Profiling Interactive Analysis 2 (GEPIA2) platform.

**FIGURE 2:**
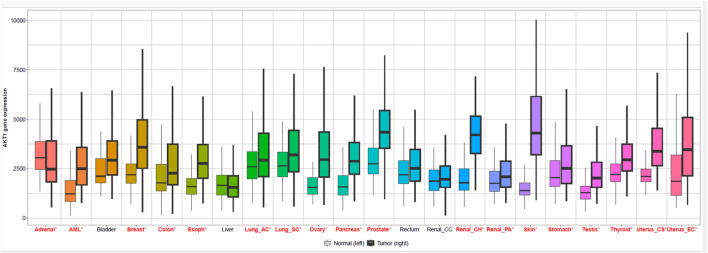
Comparison of AKT1 expression between normal and tumor tissues using TNMplot. Boxplot illustrating log2-transformed TPM values of AKT1 in normal versus tumor samples across various human cancers using the TNMplot transcriptomic dataset.

**FIGURE 3:**
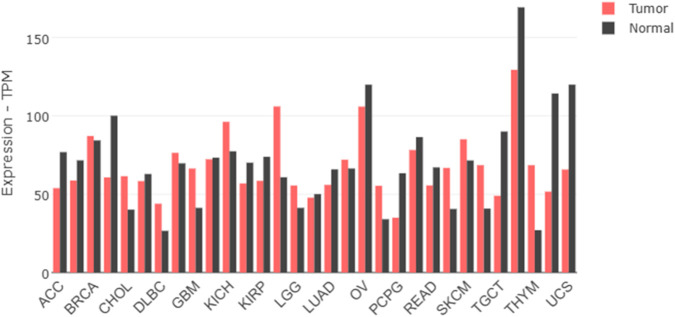
Gene expression of protein kinase B isoform 2 (AKT2) across multiple cancer types (GEPIA2). Boxplot showing differential AKT2 expression in normal (left) and tumor (right) samples across diverse cancer types. TPM: Transcripts Per Million.

**FIGURE 4:**
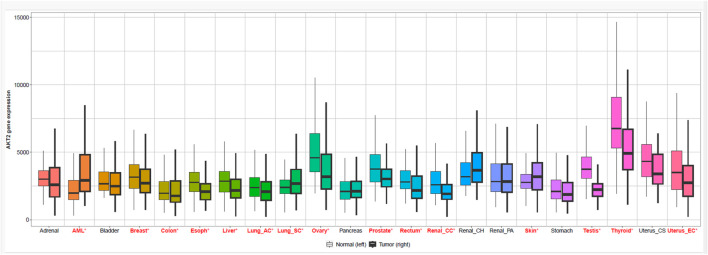
Comparison of AKT2 expression between normal and tumor tissues using TNMplot. TNMplot-based boxplot displaying log2-transformed expression values of AKT2 in normal and tumor tissues.

To establish the clinical relevance of AKT1 and AKT2 as therapeutic targets, we analyzed their differential expression across cancers using GEPIA2 and TNMplot. GEPIA2-based RNA-seq data revealed widespread overexpression of AKT1 in several tumor types, including breast (BRCA), kidney renal clear cell carcinoma (KIRC), lung adenocarcinoma (LUAD), and ovarian cancer (OV), relative to normal tissues (Figures 1, 2). Interestingly, in thymoma (THYM) and testicular germ cell tumors (TGCT), AKT1 expression was higher in normal tissue than tumor samples, suggesting a context-dependent regulation. AKT2 displayed a more tumor-specific expression pattern (Figures 3, 4). Elevated expression was noted in colorectal (COAD, READ), breast (BRCA), and ovarian (OV) tumors, while reduced levels were observed in renal cancers (KIRC, KIRP) and thymoma. TNMplot analysis corroborated these findings, showing consistent AKT1 overexpression in breast, ovary, and pancreatic tumors, while AKT2 was selectively upregulated in colorectal and ovarian cancers but downregulated in renal tumors. Collectively, the results highlight both AKT1 and AKT2 as variably dysregulated genes depending on the cancer type. AKT1 is more universally overexpressed in a wider range of tumors, whereas AKT2 shows more cancer-type-specific patterns.

### 3.2 Comparative genomic and prognostic landscape of AKT1 and AKT2 across human cancers

**FIGURE 5:**
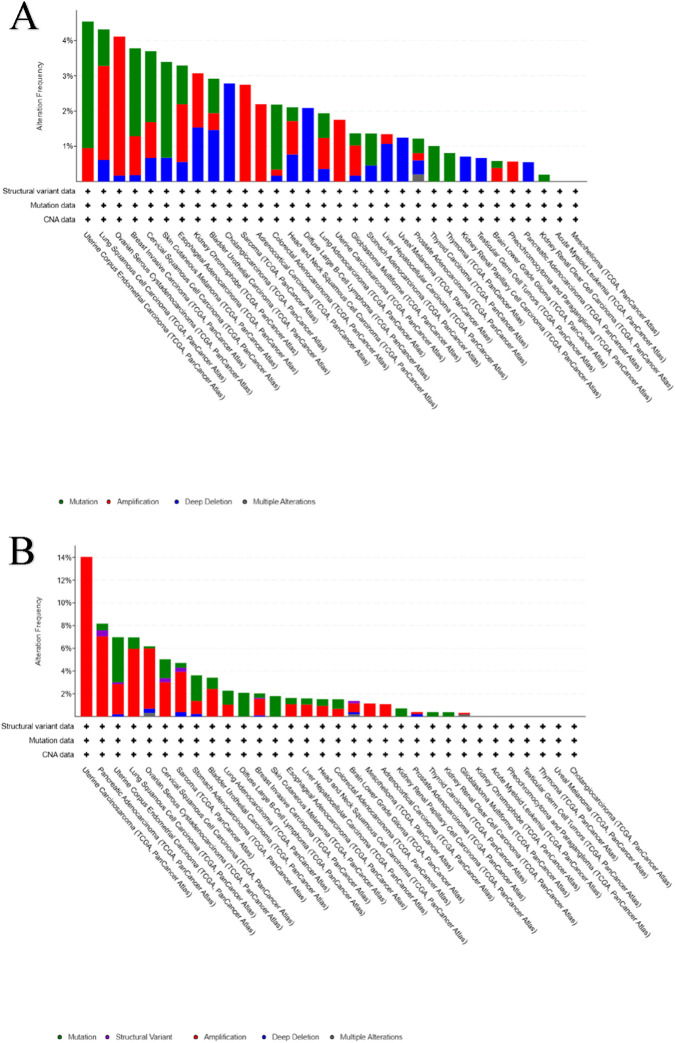
**(A,B)** Pan-cancer genomic alteration frequencies for AKT1 **(A)** and AKT2 **(B)**. Bar plots summarizing mutation, amplification, and deletion events across The Cancer Genome Atlas (TCGA) cancer types for each AKT isoform.

Comprehensive pan-cancer analysis of *AKT1* and *AKT2* using the TCGA PanCancer Atlas datasets in cBioPortal revealed distinct yet complementary genomic alteration profiles across multiple tumor types (Figures 5A,B). Both isoforms exhibited recurrent alterations, with overall alteration frequencies of ∼2.2% for AKT1 and ∼2.5% for AKT2 across all cancer cohorts. The distribution of alteration types varied markedly between the two isoforms: *AKT1* alterations were dominated by missense mutations, while *AKT2* alterations were primarily driven by copy-number amplifications (Figures 5A,B).

#### 3.2.1 Mutation distribution and structural mapping

**FIGURE 6:**
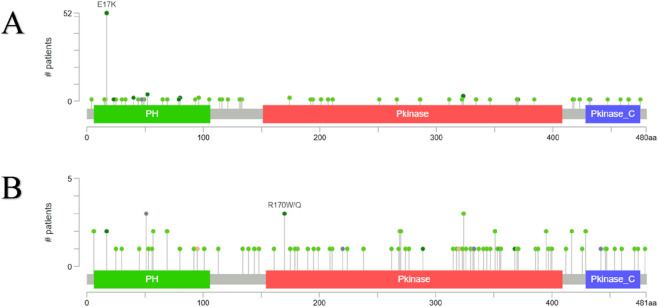
**(A,B)** Amino acid–level mutation distribution of AKT1 **(A)** and AKT2 **(B)**. Lollipop plots depicting the frequency, position, and type of missense, truncating, and other mutation categories affecting each isoform.

Lollipop plot visualization of *AKT1* revealed a predominant E17K hotspot mutation within the Pleckstrin Homology (PH) domain (Figure 6A), which accounted for over 50 cases and represents a known activating substitution that enhances membrane recruitment and constitutive signaling. Additional scattered mutations were observed throughout the kinase and C-terminal regions, suggesting limited but functionally relevant diversity. In contrast, *AKT2* displayed R170W/Q mutations in the PH domain and dispersed missense variants across the catalytic (Pkinase) and regulatory regions (Figure 6B), indicating broader domain-level plasticity and possible allosteric effects on kinase activity.

#### 3.2.2 Copy-number alterations and mRNA expression correlation

**FIGURE 7:**
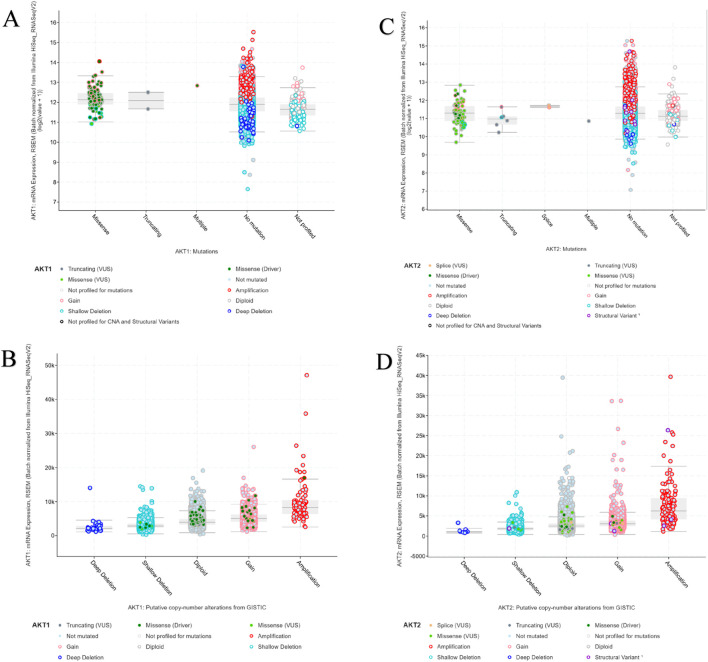
**(A–D)** Correlation of AKT1 and AKT2 mRNA expression with mutation and copy-number alteration status. Scatter and box plots showing how RSEM-normalized mRNA levels differ among samples with distinct mutation types (**(A,B)** for AKT1; **(C,D)** for AKT2).

Copy-number and mRNA expression analyses demonstrated a strong dosage effect in both isoforms (Figures 7A–D). *AKT1* amplifications corresponded with elevated mRNA expression relative to diploid and deletion states, confirming transcriptional upregulation in copy-number–altered tumors. Similarly, *AKT2* exhibited a clear expression gradient from deep deletions to amplified states, with amplification showing the highest mRNA abundance. These findings suggest gene dosage–driven overexpression as a primary mode of AKT pathway activation, especially for *AKT2*.

#### 3.2.3 Pathway-level alterations and Co-Mutational profiles

**FIGURE 8:**
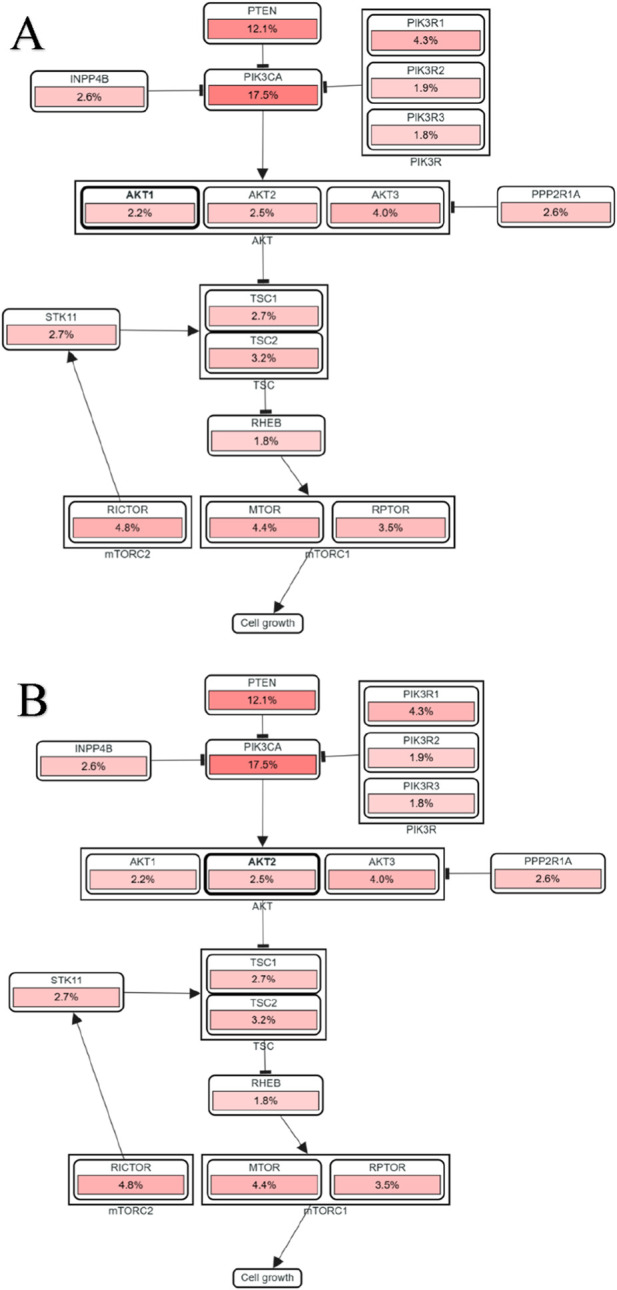
**(A,B)** Alteration map of the PI3K–AKT–mTOR pathway across cancer types. Heatmap summarizing co-alteration frequencies of key pathway regulators associated with AKT1 **(A)** and AKT2 **(B)**.

Pathway-level mapping positioned both *AKT1* and *AKT2* as central signaling nodes within the PI3K–AKT–mTOR axis, co-altered with upstream regulators such as *PIK3CA* (17.5%), *PTEN* (12.1%), and *PIK3R1* (4.3%), and downstream effectors including *MTOR* (4.4%), *RICTOR* (4.8%), and *RPTOR* (3.5%) (Figures 8A,B). These coordinated alterations reflect conserved pathway dysregulation promoting oncogenic growth and survival.

**FIGURE 9:**
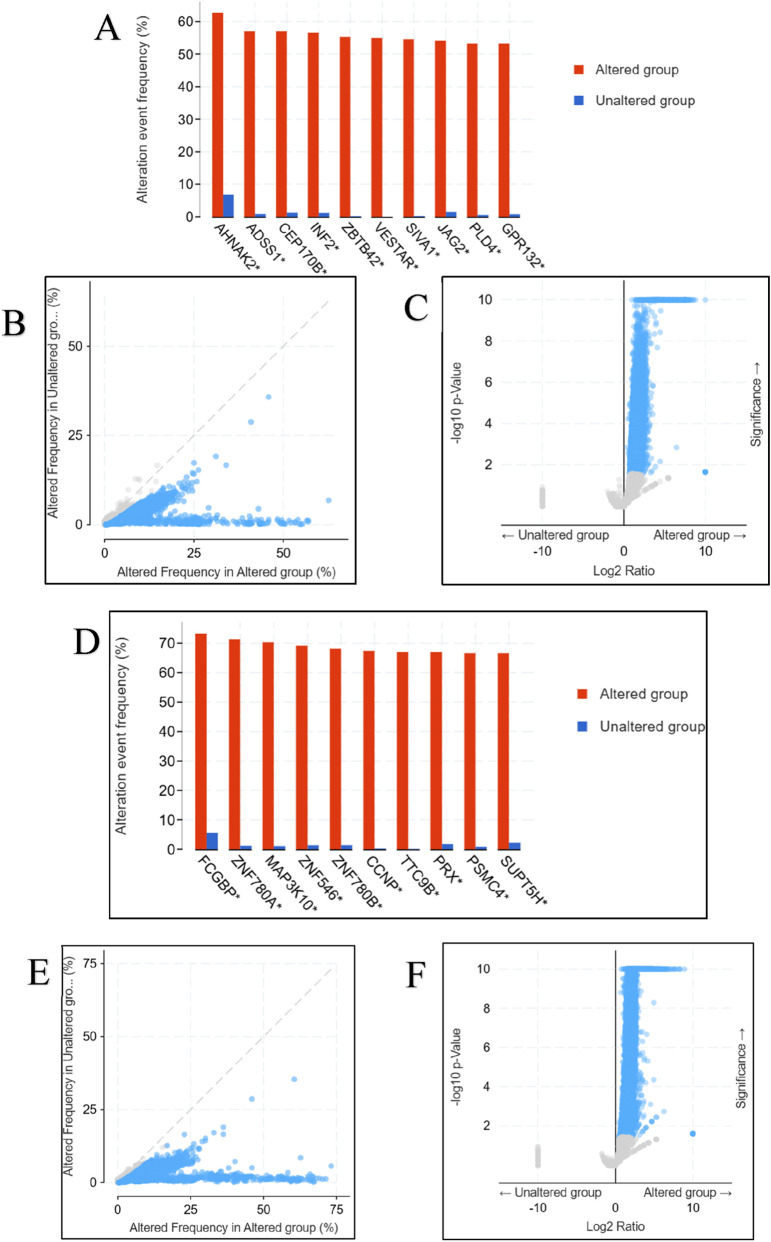
**(A–F)** Co-alteration and gene-group comparison analysis for AKT1 and AKT2. Plots highlighting the most frequently co-altered genes associated with AKT1 **(A–C)** and AKT2 **(D–F)**, including mutual exclusivity and co-occurrence statistics.

Co-occurrence and mutual exclusivity analyses identified distinct co-alteration patterns: *AKT1*-altered tumors frequently co-mutated with *AHNAK2*, *ADSS1*, *INF2*, *ZBTB42*, and *SIVA1*, whereas *AKT2*-altered tumors were enriched for co-alterations in *FCGBP*, *MAP3K10*, *ZNF780 A/B*, and *CCNP* (Figures 9A–F). These gene sets implicate divergent interaction networks, highlighting *AKT1*’s link to cytoskeletal remodeling and *AKT2*’s association with transcriptional and metabolic regulation.

#### 3.2.4 Cancer-type–specific alteration profiles

Comparative pan-cancer summary plots (Figures 4A,B) revealed distinct tumor-type enrichment patterns. *AKT1* alterations were most prevalent in endometrial, breast, cervical, and bladder carcinomas, with amplification and E17K mutation as key drivers. Conversely, *AKT2* alterations were enriched in ovarian, uterine, and colorectal cancers, predominantly via gene amplification and shallow gain events. The high frequency of *AKT2* amplifications in gynecological malignancies underscores its contribution to sustained proliferative signaling and metabolic reprogramming.

#### 3.2.5 Clinical impact on survival

**FIGURE 10:**
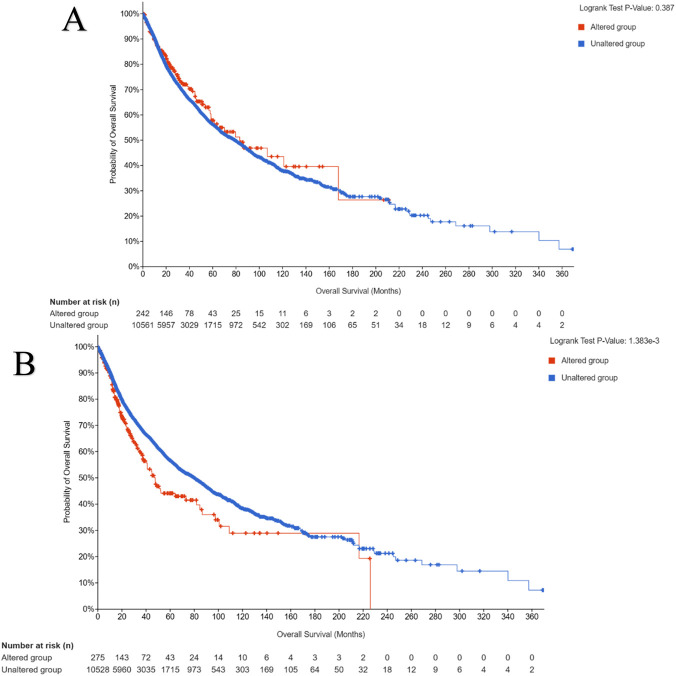
**(A,B)** Overall survival analysis for altered vs. unaltered AKT1 **(A)** and AKT2 **(B)**. Kaplan–Meier plots comparing survival outcomes in patients stratified by genomic alteration status of AKT1 or AKT2.

Survival analysis demonstrated a significant prognostic distinction between the two isoforms (Figures 10A,B). *AKT1*-altered patients showed no statistically significant difference in overall survival compared to unaltered cases (*P* = 0.387), whereas *AKT2* alterations correlated with markedly reduced survival (*P* = 1.38 × 10^−3^), indicating that *AKT2* amplification or mutation status may serve as a negative prognostic marker in diverse cancers.

#### 3.2.6 Prognostic correlation of AKT1 and AKT2 expression

**FIGURE 11:**
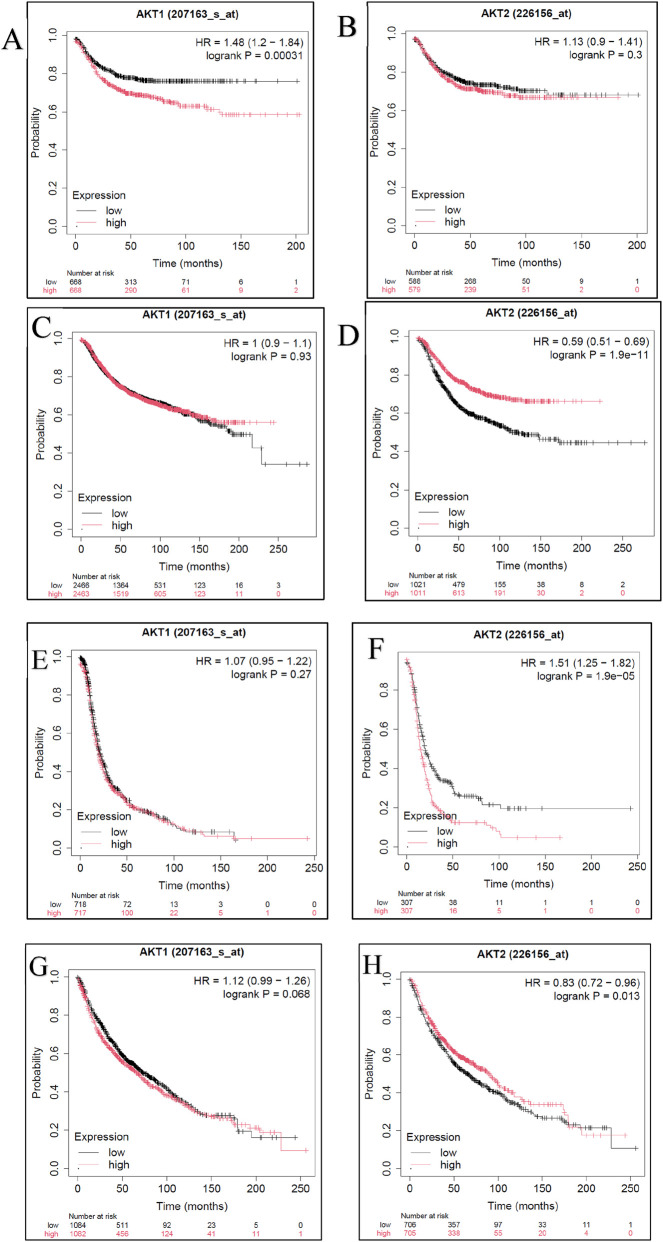
**(A–H)** Cancer-type–specific survival associations of AKT1 and AKT2 expression. Kaplan–Meier survival plots for colon **(A)**, breast **(C)**, ovarian **(E)**, and lung **(G)** cancers, stratified by high versus low mRNA expression of AKT1. Kaplan–Meier survival plots for colon **(A)**, breast **(C)**, ovarian **(E)**, and lung **(G)** cancers, stratified by high versus low mRNA expression of AKT2.

Survival analysis performed using the Kaplan–Meier Plotter revealed cancer-type–specific prognostic associations of *AKT1* and *AKT2* expression (Figures 11A–H). In colon carcinoma, *AKT1* overexpression was significantly correlated with poor overall survival (HR = 1.48, 95% CI 1.20–1.84; *P* = 0.00031), whereas *AKT2* showed no significant association (*P* = 0.30). Conversely, in breast cancer, *AKT2* overexpression was associated with a markedly improved prognosis (HR = 0.59, 95% CI 0.51–0.69; *P* = 1.9 × 10^−11^), while *AKT1* expression had no effect (*P* = 0.93). In ovarian cancer, *AKT2* amplification correlated with reduced patient survival (HR = 1.51, 95% CI 1.25–1.82; *P* = 1.9 × 10^−5^), indicating its potential role as a prognostic biomarker. Lung cancer datasets displayed a mild, non-significant trend toward decreased survival with elevated *AKT1* (HR = 1.12; *P* = 0.068) but modestly improved outcomes with higher *AKT2* levels (HR = 0.83; *P* = 0.013). Overall, these results emphasize the isoform-specific and tumor-context–dependent prognostic behavior of AKT kinases, reinforcing the need for selective inhibition strategies explored in this study.

### 3.3 Molecular docking and binding affinity evaluation

**FIGURE 12:**
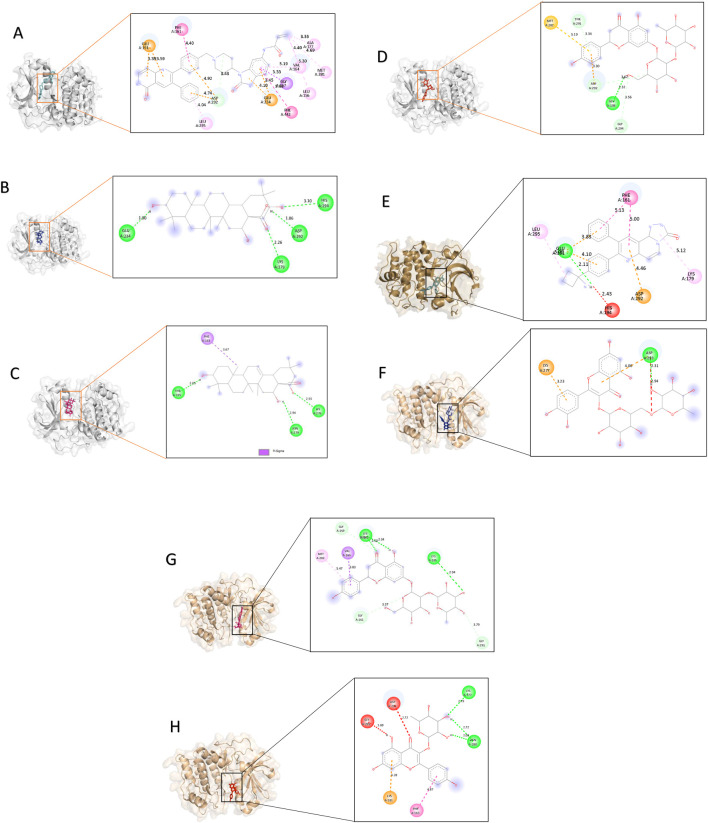
Molecular docking poses of the top phytochemicals with AKT1 and AKT2. Panels **(A–D)** show binding interactions of the control inhibitor, oleanolic acid, pitheduloside I, and naringin with AKT1 (PDB ID: 3QKL). Panels **(E–H)** show corresponding docking poses within the AKT2 binding site (PDB ID: 2JDO). Hydrogen-bonding and hydrophobic contacts are highlighted.

A library of 20 phytochemicals from *P. dulce* was docked against AKT1 (PDB: 3QKL) and AKT2 (PDB: 2JDO) to explore isoform-selective inhibitory potential. The docking results were benchmarked against known AKT inhibitors MK-2206 for AKT1 and Ipatasertib for AKT2 as control compounds. Among these, oleanolic acid, pitheduloside I, naringin, and rutin emerged as top-scoring ligands, demonstrating the lowest binding energies and highest affinities (Tables 1, 2). Specifically, oleanolic acid exhibited the a more favorable docking score to AKT1 with a docking energy of −15.0 kcal/mol, and with the control inhibitor MK-2206 (−11.2 kcal/mol). For AKT2, rutin displayed the highest binding affinity with an energy of −10.8 kcal/mol, comparable to the reference inhibitor Ipatasertib (−10.1 kcal/mol). Pitheduloside I and naringin also demonstrated favorable binding toward AKT1 and AKT2, respectively, with docking energies ranging from −9.8 to −11.3 kcal/mol. Figure 12 presents the docked conformations of the top three ligands and control compounds within the binding pockets of each isoform, highlighting distinct residue interactions.

**TABLE 1:** Binding energies (kcal/mol) of Pithecellobium dulce phytochemicals against AKT1 predicted by AutoDock Vina.

Compound	Chem ID	Binding energy (kcal/mol)
Afzelin	5316673	−9.8
Campesterol	173183	−8.9
Catechol	289	−5.4
Coumaric acid	637542	−6
Daidzein	5281708	−8.3
Echinocystic acid	73309	−10.1
Ellagic acid	5281855	−8.8
Ferulic acid	445858	−6
Galacitol	11850	-.6.6
Gallic acid	370	−5.9
Hederagenin	73299	−10.3
Kaempferol	5280863	−8.5
Leucofisetinidin	12305025	−7.9
Mandelic acid	1292	−5.8
Melacacidin	169996	−8.2
Naringin	442428	−10.4
Octacosanol	68406	−5
Oleanolic acid	10494	−15
Pinitol	164619	−5.7
PithedulosideI	12305892	−11.3
Pitheduloside I	12305892	−6.2
Quercetin	5280343	−7.7
Rutin	5280805	−10.1
Stigmasterol	5280794	−7.9
Vanillic acid	8468	−5.6
α-spinasterol	482043048	−9.2
Control inhibitor MK-2206	24964624	−11.2

**TABLE 2:** Binding energies (kcal/mol) of Pithecellobium dulce phytochemicals against AKT2 predicted by AutoDock Vina.

Compound	Chem ID	Binding energy (kcal/mol)
Afzelin	5316673	−9.5
Campesterol	173183	−8.5
Catechol	289	−5.7
Coumaric acid	637542	−6.1
Daidzein	5281708	−8.3
Echinocystic acid	73309	−8.9
Ellagic acid	5281855	−8.8
Ferulic acid	445858	−6.4
Galacitol	11850	−5.1
Gallic acid	370	−6.2
Hederagenin	73299	−7.8
Kaempferol	5280863	−8.9
Leucofisetinidin	12305025	−8.2
Mandelic acid	1292	−6.1
Melacacidin	169996	−8.8
Naringin	442428	−9.8
Octacosanol	68406	−4.9
Oleanolic acid	10494	−9.4
Pinitol	164619	−5.8
Pitheduloside I	12305892	−7.5
Quercetin	5280343	−9
Rutin	5280805	−10.8
Stigmasterol	5280794	−8
Vanillic acid	8468	−6
α-spinasterol	482043048	−8.9
Control inhibitor ipatasertib	24788740	−9.1

### 3.4 Protein-ligand interaction mapping

Protein-ligand interface analyses identified key binding residues and characteristic interaction patterns for each isoform-ligand combination. In AKT1 complexes, oleanolic acid, pitheduloside I, and naringin established strong hydrogen bonds and notable π-interactions with Glu234, Asp292, Lys179, and His194, with these contacts remaining highly conserved and persistent throughout the simulations. In contrast, AKT2 complexes, particularly with rutin and naringin, primarily engaged Lys160, Thr162, Asp293, and Met229, exhibiting a broader but comparatively less focused and persistent interaction profile than AKT1. Table 3 details the interaction types, bond distances, and binding energies, highlighting the critical role of Glu and Asp residues in anchoring and stabilizing the ligands.

**TABLE 3:** Key molecular interactions and MM-GBSA binding energies for oleanolic acid, pitheduloside I, and naringin in complex with AKT1 and AKT2. Interaction residues and interaction types (hydrogen bonds, hydrophobic contacts) are listed.

Protein	Ligand	Interacting residues	Type of interaction	Distance
AKT1	MK-2206	Asp 292 Phe 442 Gly 157 Glu 234	Carbon hydrogen bond Pi-Pi stacked Pi-Sigma Pi-Anion	Asp 3.43 Phe 3.45 Gly 3.33 Glu 4.10
	Oleanolic acid	Glu 234 Asp 292 Lys 179 His 194	Hydrogen bond	Glu 1.90 Asp 1.86 Lys 2.26 His 3.10
	Pitheduloside I	Thr 195 Phe 161 Lys 276 Asn 279	Hydrogen bond Sigma (Phe)	Thr 2.05 Phe 3.67 Lys 2.55 Asn 2.94
	Naringin	Met 281 Thr 291 Asp 292 Glu 198 Gly294	Hydrogen bond (Glu) Carbon hydrogen bond (Gly, Asp) Pi anion, sulphur (met) Pi donor hydrogen bond (Thr)	Met 5.19 Thr 3.34 Asp 3.30 Glu 2.32 Gly 3.56
AKT2	Ipatasertib	Glu 191 Asp 292 His 194 Lys 179	Hydrogen bond Pi anion Unfavorable donor Pi-Alkyl	Glu 2.11 Asp 4.46 His 2.43 Lys 5.12
	Rutin	Glu 234 Asp 292 Lys 179 His 194	Hydrogen bond	Glu 1.90 Asp 1.86 Lys 2.26 His 3.10
	Naringin	Thr 195 Phe 161 Lys 276 Asn 279	Hydrogen bond Sigma (Phe)	Thr 2.05 Phe 3.67 Lys 2.55 Asn 2.94
	Afzelin	Met 281 Thr 291 Asp 292 Glu 198 Gly294	Hydrogen bond (Glu) Carbon hydrogen bond (Gly, Asp) Pi anion, sulphur (met) Pi donor hydrogen bond (Thr)	Met 5.19 Thr 3.34 Asp 3.30 Glu 2.32 Gly 3.56

### 3.5 ADME and drug-likeness properties

The phytochemicals were further assessed for drug-likeness and ADME profiles using SwissADME and evaluated against Lipinski’s rule of five. Oleanolic acid and naringin fully satisfied all Lipinski criteria for oral bioavailability, whereas rutin met four out of five parameters, with the exception being its higher molecular weight. Most of the evaluated candidates exhibited favorable cLogP values, hydrogen bond donor and acceptor counts, and molar refractivity, indicating physicochemical properties conducive to drug development. Collectively, these results suggest a strong potential for oral absorption and favorable pharmacokinetic attributes, consistent with the profiles of established natural product–derived drug leads. Complete ADME and physicochemical data are provided in Table 4.

**TABLE 4:** Physicochemical and ADME (absorption, distribution, metabolism, excretion) properties of selected P. dulce phytochemicals derived from SwissADME.

Molecule	Molecular weight (g/mol)	ESOL log S	ESOL class	GI absorption	BBB permeant	Log Kp (cm/s)	Lipinski #violations	Bioavailability score
Afzelin	432.38	−3.47	Soluble	Low	No	−8.07	Yes	0.55
Campesterol	400.68	−7.54	Poorly soluble	Low	No	−2.5	Yes	0.55
Catechol	110.11	−1.63	Very soluble	High	YES	−6.35	Yes	0.55
Coumaric acid	164.16	−2.02	Soluble	High	YES	−6.26	Yes	0.85
Daidzein	254.24	−3.53	Soluble	High	yes	−6.1	Yes	0.55
Echinocystic acid	472.70	−7.04	Poorly soluble	High	No	−4.3	Yes	0.56
Ellagic acid	302.19	−2.94	Soluble	High	No	−7.36	Yes	0.55
Ferulic acid	194.18	−2.11	Soluble	High	YES	−6.41	Yes	0.85
Galactitol	182.17	1.31	Highly soluble	Low	No	−9.61	Yes	0.55
Gallic acid	170.12	−1.64	Very soluble	High	No	−6.84	Yes	0.56
Hederagenin	472.70	−6.94	Poorly soluble	High	No	−4.34	Yes	0.56
Kaempferol	286.24	−3.31	Soluble	High	No	−6.7	Yes	0.55
Leucofisetinidin	290.27	−2.41	Soluble	High	No	−7.6	Yes	0.55
Mandelic acid	152.15	−1.45	Very soluble	High	No	−6.79	Yes	0.85
Melacacidin	306.27	−2.27	Soluble	High	No	−7.95	Yes	0.55
Naringin	580.53	−2.98	Soluble	Low	No	−10.15	No	0.17
Octacosanol	410.76	−9.25	Poorly soluble	Low	No	0.86	Yes	0.55
Oleanolic acid	456.70	−7.32	Poorly soluble	Low	No	−3.77	Yes	0.85
Pinitol	194.18	1.02	Highly soluble	Low	No	−9.74	Yes	0.55
PithedulosideI	488.7	−6.29	Poorly soluble	High	No	−5.35	Yes	0.56
Pitheduloside E	899.07	−5.94	Moderately soluble	Low	No	−10.59	No	0.11
Pitheduloside G	1045.21	−6.03	Poorly soluble	Low	No	−12.19	No	0.11
Pitheduloside K	1061.21	−5.74	Moderately soluble	Low	No	−12.72	No	0.11
Quercetin	302.24	−3.16	Soluble	High	No	−7.05	Yes	0.55
Rutin	610.52	−3.3	Soluble	Low	No	−10.26	No	0.17
Stigmasterol	412.69	−7.46	Poorly soluble	Low	No	−2.74	Yes	0.55
Vanillic acid	168.15	−2.02	Soluble	High	No	−6.31	Yes	0.85
α-spinasterol	412.69	−7.3	Poorly soluble	Low	No	−2.92	Yes	0.55

### 3.6 DFT quantum chemical analysis

**FIGURE 13:**
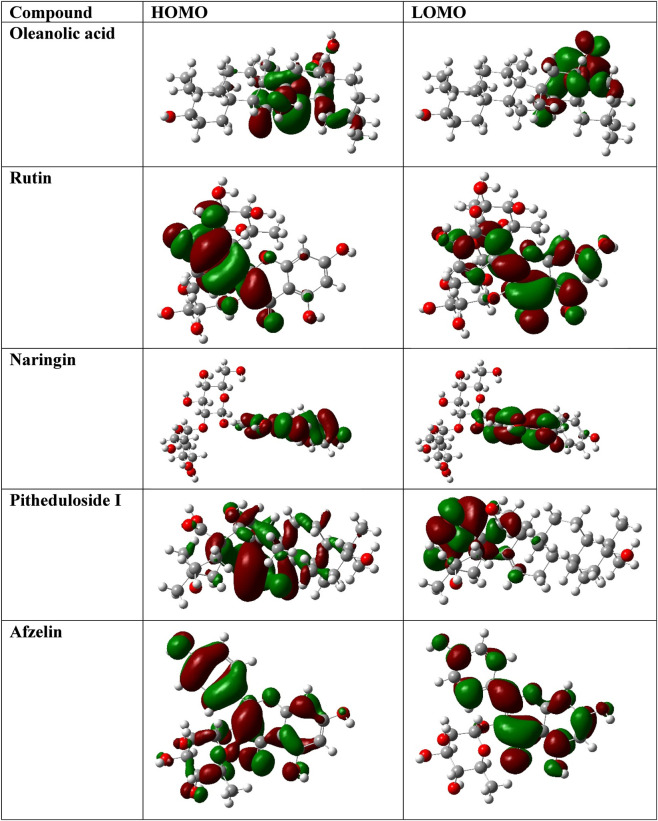
Frontier molecular orbitals (FMOs) of top-scoring *Pithecellobium dulce* phytochemicals. Highest occupied molecular orbital (HOMO) and lowest unoccupied molecular orbital (LUMO) representations (left and right panels for each compound) plotted at identical isosurface values. Red and green regions represent opposite orbital phases.

DFT calculations (RB3LYP/6-31G (d,p)) revealed distinct electronic profiles for the top *P. dulce* phytochemicals (Table 5). Oleanolic acid (−1397.8401 a.u.; 2.92 D) and naringin (−2101.1775 a.u.; 1.10 D) showed low-to-moderate polarity, supporting stable hydrophobic anchoring in AKT1. Rutin exhibited the highest dipole moment (−2250.3651 a.u.; 7.98 D), favoring dynamic polar interactions with AKT2. Afzelin and pitheduloside I had intermediate polarity, enabling mixed hydrophobic–polar binding. FMO maps (Figure 13) indicated HOMO localization on hydrophobic cores and LUMO on polar regions for low-polarity ligands, while high-polarity ligands displayed delocalized orbitals over polyhydroxylated regions, correlating with their binding adaptability.

**TABLE 5:** Quantum-chemical descriptors (total energy and dipole moment) of top-scoring *P. dulce* phytochemicals and their implications for predicted AKT isoform selectivity.

Compound	Total energy (a.u.)	Dipole moment (Debye)
Oleanolic acid	−1397.8401	2.92
Rutin	−2250.3651	7.98
Naringin	−2101.1775	1.10
Pitheduloside I	−1547.8441	4.29
Afzelin	−1564.3719	5.33

### 3.7 Molecular dynamics simulation and stability analysis

To gain mechanistic insights into the dynamic stability and conformational flexibility of the AKT–ligand systems, 200 ns molecular dynamics (MD) simulations were carried out for both the control (native co-crystallized inhibitors) and phytochemical ligand complexes (oleanolic acid–AKT1 and rutin–AKT2). The analyses, including RMSD, RMSF, and protein–ligand contact mapping, provided a comparative understanding of structural integrity and binding persistence across the two isoforms.

#### 3.7.1 RMSD analysis

**FIGURE 14:**
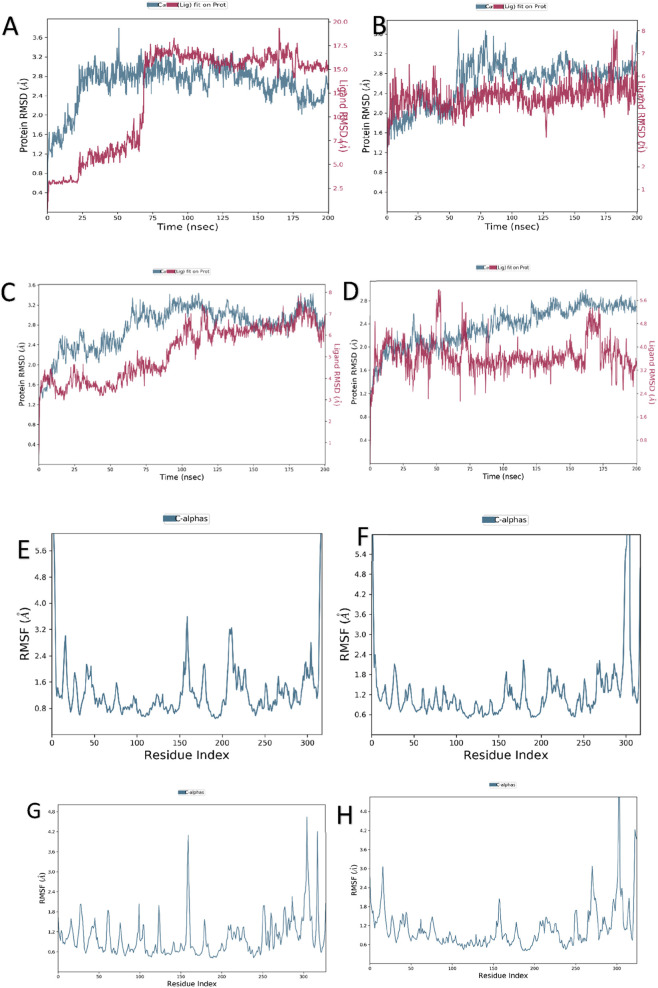
**(A–H)** Molecular dynamics (MD) simulation analysis of AKT1 and AKT2 complexes. **(A,B)** Root-mean-square deviation (RMSD) profiles showing structural stability of AKT1 with Control and Oleanolic acid. **(C,D)** Root-mean-square deviation (RMSD) profiles showing structural stability of AKT2 with Control and Rutin. **(E–F)** Root-mean-square fluctuation (RMSF) profiles of AKT1 with Control and Oleanolic acid highlighting residue-wise flexibility during the simulation. **(G–H)** Root-mean-square fluctuation (RMSF) profiles of AKT2 with Control and Rutin highlighting residue-wise flexibility during the simulation.

The Cα RMSD plots revealed that the AKT1 control complex-maintained backbone stability with fluctuations between 1.5 and 2.5 Å, while the ligand RMSD ranged from 1.0 to 3.5 Å, suggesting robust complex integrity (Figure 14A). Similarly, the oleanolic acid–AKT1 complex exhibited a well-equilibrated trajectory with RMSD values ranging from 2.0 to 2.8 Å, indicating a slightly flexible yet structurally stable binding environment (Figure 14B). In contrast, the AKT2 control complex displayed moderate protein fluctuations between 2.0 and 3.0 Å and ligand deviations up to 6.0 Å (Figure 14C), whereas the rutin–AKT2 complex showed greater dynamic variability, with protein RMSD reaching 3.6 Å and ligand RMSD extending up to 7.0 Å (Figure 14D). Both systems reached equilibrium after approximately 75 ns, confirming overall conformational convergence.

#### 3.7.2 RMSF analysis

The residue-wise RMSF analysis provided further insights into local flexibility changes (Figures 14E–H). For AKT1, both the control and oleanolic acid complexes demonstrated minimal fluctuations (<1.5 Å) in key catalytic residues within the binding pocket, with only minor mobility observed in the terminal and loop regions. Conversely, AKT2 showed higher fluctuations overall, with the rutin complex displaying enhanced flexibility (2.5–4.8 Å) in loop and substrate-binding regions compared to its control. This increased mobility indicates a more adaptable and solvent-exposed binding groove, likely influenced by the polar characteristics of rutin.

#### 3.7.3 Protein–ligand contact analysis

**FIGURE 15:**
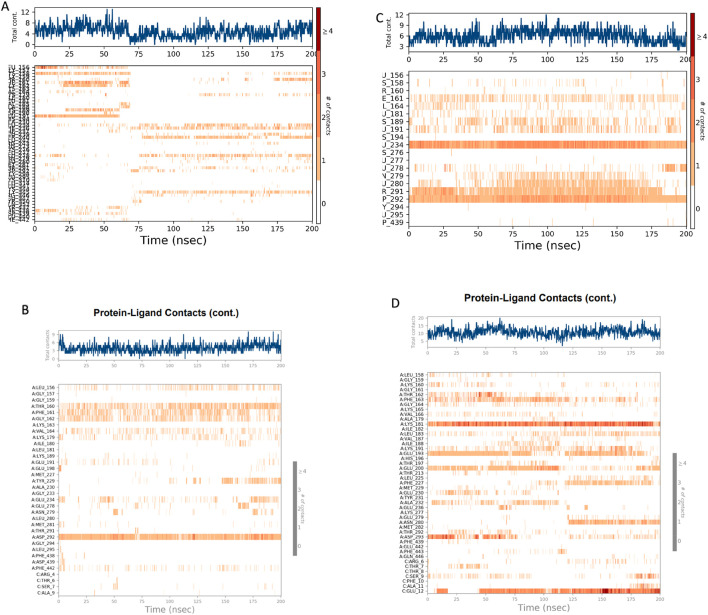
**(A–D)** Protein–ligand interaction frequency maps from MD simulations. **(A,B)** Contact maps showing the persistence and frequency of hydrogen bonds, hydrophobic contacts, and water bridges between control ligand and Oleanolic acid. **(C,D)** Contact maps showing the persistence and frequency of hydrogen bonds, hydrophobic contacts, and water bridges between control ligand and Rutin.

**FIGURE 16:**
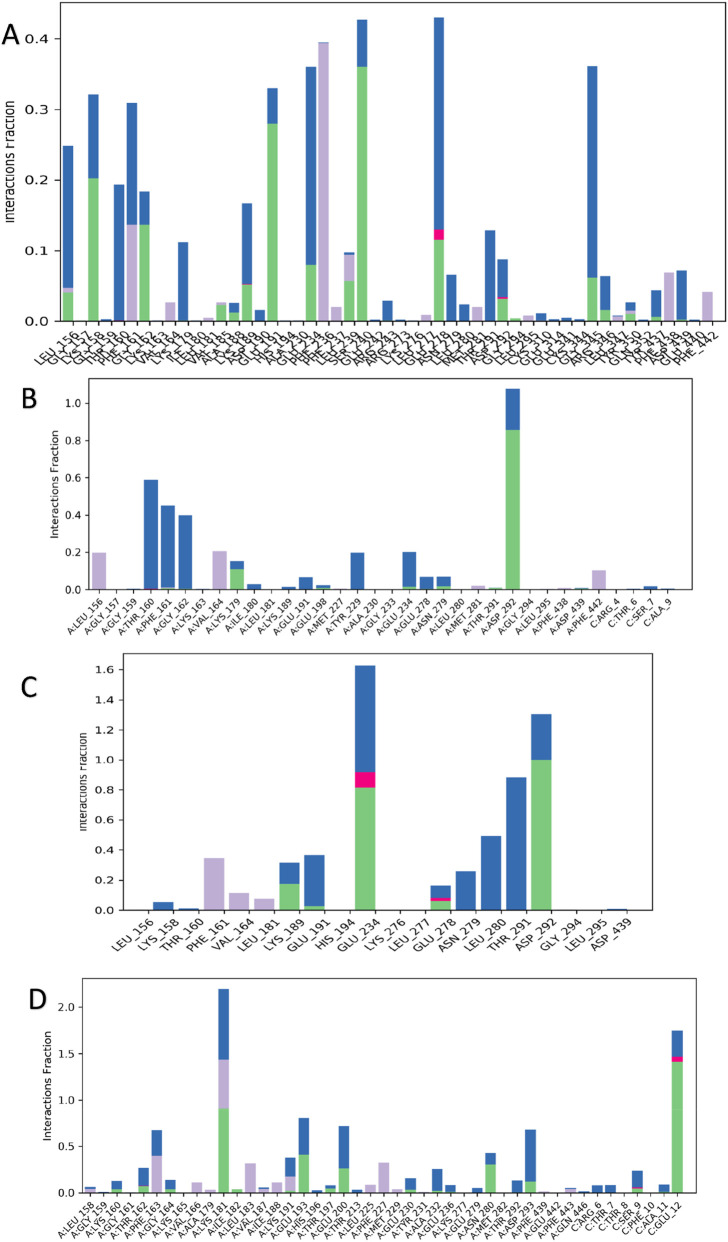
**(A–D)** Histogram of protein–ligand contact persistence. **(A,B)** Quantitative representation of the number and type of interactions formed by control ligand and Oleanolic acid with AKT1 and **(C,D)** Quantitative representation of the number and type of interactions formed by control ligand and Rutin with AKT2 throughout the MD simulation.

Protein–ligand interaction timelines (Figures 15A–D) and contact histograms (Figures 16A–D) revealed clear differences in binding persistence. The AKT1 control complex maintained strong and stable contacts with LEU156, THR160, ASP292, and GLY294, persisting for most of the trajectory. Similarly, the oleanolic acid–AKT1 complex retained high-occupancy interactions with ALA157, ALA162, and ALA292, indicating durable hydrogen-bond and hydrophobic interactions throughout the simulation.

In the case of AKT2, the control complex exhibited several short-lived contacts, predominantly involving LYS160, THR162, ASP293, and MET229, whereas the rutin–AKT2 complex demonstrated more distributed and transient interactions with THR162, ASP293, and GLY229, reflecting dynamic binding but lower contact persistence. These results collectively highlight stronger ligand retention and compact binding in AKT1 compared to the more flexible, fluctuating pattern in AKT2.

#### 3.7.4 Comparative interpretation

Integrative evaluation of RMSD, RMSF, and interaction profiles clearly demonstrates isoform-selective stability between the phytochemicals and AKT isoforms. The oleanolic acid–AKT1 complex exhibited dynamic behavior highly comparable to its control complex, maintaining a compact protein structure and persistent hydrophobic interactions, suggesting effective mimicry of the native inhibitor’s stabilization pattern. In contrast, the rutin–AKT2 complex displayed elevated conformational fluctuations relative to its control, reflecting an adaptive binding mode facilitated by rutin’s high polarity and larger size.

Overall, these findings indicate that oleanolic acid binds more tightly and stably to AKT1, favoring hydrophobic anchoring within a rigid binding cavity, whereas rutin preferentially interacts with AKT2 through flexible, transient hydrogen-bonding networks. The correspondence between MD behavior and quantum chemical descriptors supports the conclusion that moderate polarity favors compact, stable binding (AKT1–oleanolic acid), while higher polarity promotes dynamic, reversible interactions (AKT2–rutin).

Collectively, the integration of docking, pharmacokinetic profiling, electronic characterization, and MD simulations reveals that *Pithecellobium dulce*–derived phytocompounds exhibit distinct isoform-specific binding patterns with oleanolic acid emerging as a potential AKT1-selective inhibitor and rutin as an AKT2-preferential modulator providing a rational foundation for future structure-guided drug design and experimental validation in cancer signaling models.

### 3.8 Binding free energy estimation by MM-GBSA

MM-GBSA calculations using the Prime module (Schrödinger 2023-1) estimated average binding free energies (ΔG_bind) from the final 50 ns of MD trajectories. The oleanolic acid–AKT1 complex showed a highly favorable ΔG_bind of −68.4 kcal/mol (±4.2), driven mainly by van der Waals and lipophilic interactions, consistent with its stable binding observed in docking and MD. In comparison, the rutin–AKT2 complex had a less favorable ΔG_bind of −52.7 kcal/mol (±5.1), with binding dominated by electrostatic and polar contributions, reflecting its more flexible and dynamic interaction profile.

## 4 Discussion

The PI3K–AKT–mTOR signaling pathway is central to regulating cellular processes such as cell growth, metabolism, survival, and migration. Dysregulation of this pathway, particularly through mutations or amplifications of AKT isoforms, is a hallmark of various cancers, contributing to tumor progression, metastasis, and resistance to therapies. Our study, which explored the isoform-selective inhibition of AKT1 and AKT2 using *P. dulce* phytochemicals, highlights the potential for targeted cancer therapies with reduced off-target effects and improved selectivity.

From a pharmacogenomic standpoint, the distinct genetic alterations in AKT1 and AKT2 such as activating E17K mutation versus copy-number amplification represent molecular signatures that can guide patient-specific therapeutic strategies (Cecchin and Stocco, 2020). Integrating such genomic insights with *in silico* inhibitor screening offers a rational approach to precision oncology.

### 4.1 Isoform-specific roles of AKT in cancer

AKT1 and AKT2 play distinct roles in cancer biology, and their dysregulation has been linked to different cancer types. AKT1 is primarily involved in regulating cell survival and proliferation. Overexpression of AKT1 is frequently observed in various cancers, including breast, ovarian, and colorectal cancers (Degan and Gelman, 2021; Adon et al., 2025). The E17K mutation, a well-known activating mutation in AKT1, has been found to enhance its kinase activity and is commonly seen in breast and colon cancers, leading to increased cell survival and proliferation (Gao et al., 2021; Hassan et al., 2024).

In contrast, AKT2 is more involved in regulating metabolic processes and is frequently amplified in aggressive cancers such as ovarian, pancreatic, and endometrial cancers. AKT2 amplification correlates with poor prognosis and increased metastatic potential, particularly in ovarian cancer (Tewari et al., 2022; Paul et al., 2025). These findings are in agreement with our results, which show that AKT1 is broadly dysregulated in a range of cancers, while AKT2 alterations are more cancer-type-specific, with amplification of AKT2 being associated with poor prognosis in cancers like ovarian and colorectal.

The distinct roles of AKT1 and AKT2 in cancer progression underscore the need for isoform-specific targeting strategies to reduce side effects and improve therapeutic outcomes. Previous research has shown that targeting AKT1 selectively may offer a therapeutic benefit in cancers where AKT1 plays a major role, while AKT2-specific inhibition could be critical for treating cancers where AKT2 amplification is prominent, such as ovarian and pancreatic cancers (Brown and Banerji, 2017; Fakhri et al., 2021).

Furthermore, several microRNAs and long non-coding RNAs have been reported to modulate AKT isoform activity miR-149-5p and miR-29b suppress AKT1 translation, whereas lncRNAs such as HOTAIR and MALAT1 enhance AKT2 signaling (Selvakumar S. C. et al., 2023; Xiao et al., 2024). These findings suggest that ncRNAs can influence AKT-driven oncogenesis and therapeutic sensitivity, offering an additional layer of pharmacogenomic regulation (Liu et al., 2016). The interplay between these ncRNAs and AKT isoforms may partly determine therapeutic response, emphasizing their importance in future biomarker-driven studies.

### 4.2 Integration of genomic alterations with structural interpretation

The genomic alterations identified in this study, particularly the AKT1 E17K hotspot mutation and AKT2 R170Q/W variants, provide important context for interpreting the structural behavior of the isoforms. These mutations occur within the PH domain and are known to influence membrane association, domain orientation, and accessibility of the ligand-binding region. Although the present simulations were performed using wild-type AKT1 and AKT2 structures, the differential interaction profiles observed more compact and hydrophobic binding in AKT1 versus more flexible, solvent-exposed binding in AKT2 align with the functional consequences reported for these mutations. This conceptual linkage supports the biological relevance of the isoform-selective binding patterns predicted in our computational analysis.

### 4.3 Challenges with Pan-AKT inhibitors

Traditional pan-AKT inhibitors, such as MK-2206 and Ipatasertib, have demonstrated some clinical efficacy but are limited by a lack of isoform specificity, leading to undesirable off-target effects like hyperglycemia, acneiform rashes, and liver toxicity (Lin et al., 2013; Saura et al., 2017; Harun et al., 2023). Additionally, the emergence of drug resistance through mutations in the AKT binding site or activation of compensatory survival pathways limits the long-term efficacy of these inhibitors (He et al., 2021; Savill et al., 2022).

In this context, isoform-selective AKT inhibitors offer a promising solution to these challenges. By targeting only, the specific AKT isoform implicated in cancer progression, isoform-selective inhibitors can minimize off-target effects while providing more precise therapeutic benefits. Isoform-selective AKT inhibitors have shown promising results in preclinical models, where AKT1 inhibitors reduce cell proliferation in breast cancer, and AKT2-selective inhibitors reduce metastasis in ovarian cancer (Fakhri et al., 2021; Adon et al., 2025).

Our results also highlight the potential of natural products, such as those derived from *Pithecellobium dulce*, to offer isoform-selective inhibition. The phytochemicals oleanolic acid and rutin, identified as selective inhibitors of AKT1 and AKT2, respectively, could serve as viable alternatives to current pan-AKT inhibitors (Selvakumar M. et al., 2023). These compounds exhibited binding energies comparable to or better than those of known AKT inhibitors, MK-2206 and Ipatasertib, in our molecular docking studies. This aligns with previous findings that natural products, including oleanolic acid, rutin, and other flavonoids, can selectively inhibit AKT isoforms and show anticancer activity (Dhanisha et al., 2022; Asiamah et al., 2023; Adon et al., 2025; Paul et al., 2025).

### 4.4 Comparison of phytochemical inhibitors with existing AKT inhibitors

Our molecular docking and dynamics simulation results indicate that oleanolic acid binds strongly to AKT1, with a binding energy of −15.0 kcal/mol, surpassing the known AKT1 inhibitor MK-2206 (−11.2 kcal/mol). Similarly, rutin showed a comparable binding affinity to Ipatasertib for AKT2, with a binding energy of −10.8 kcal/mol, matching the performance of the clinical inhibitor (−10.1 kcal/mol). These findings support the possibility that *P. dulce* phytochemicals may act as isoform-selective AKT binders, although experimental studies are required for validation (Dhanisha et al., 2022; Jamshidi et al., 2025; Vargas-Madriz et al., 2025). The accuracy of the docking procedure was supported by the redocking results, which reproduced the crystallographic ligand conformations with RMSD values in the acceptable range (0-2 Å), indicating that the predicted poses for the phytochemicals are consistent with experimentally observed AKT–ligand interactions.

Furthermore, the results of our molecular dynamics simulations showed that the AKT1–oleanolic acid complex maintained high stability with minimal RMSD fluctuations, suggesting that oleanolic acid forms strong, persistent interactions with AKT1. In contrast, the AKT2–rutin complex exhibited more flexibility, reflecting the dynamic binding nature of AKT2. These findings are consistent with the previously described differences in the stability and flexibility of AKT1 and AKT2 complexes (Jamshidi et al., 2025; Paul et al., 2025) Taken together, the combination of validated docking, long-timescale MD simulations, and MM-GBSA binding-energy estimation provides a rigorous and internally consistent computational framework for evaluating isoform-selective AKT binding.

### 4.5 ADME and drug-likeness of *Pithecellobium dulce* phytochemicals

The ADME analysis of oleanolic acid and rutin indicated favorable drug-likeness profiles, with both compounds meeting key Lipinski’s Rule of Five criteria for oral bioavailability. These compounds demonstrated favorable cLogP values, hydrogen bond donor and acceptor counts, and molecular weight, suggesting their potential for oral absorption and bioavailability *in vivo*. This is consistent with previous studies that have highlighted the pharmacokinetic properties of natural compounds like oleanolic acid and rutin (Author, 2025; Jamshidi et al., 2025).

### 4.6 Study scope and interpretation

The present work is a computational pharmacology study integrating genomic analysis, docking, molecular dynamics, and MM-GBSA approaches to characterize isoform-selective AKT inhibition. Accordingly, the findings represent mechanistic predictions generated through validated *in silico* methods. While they provide a strong rationale for prioritizing specific phytochemicals for downstream laboratory testing, they should be interpreted as computational evidence rather than experimental confirmation.

### 4.7 Future directions

The findings from this study provide a strong foundation for further experimental validation of the isoform-specific AKT inhibitors identified from *Pithecellobium dulce*. Future studies should focus on preclinical *in vitro* and *in vivo* models to evaluate the efficacy of these phytochemicals in cancer therapy. Moreover, combination therapy approaches that integrate these isoform-selective inhibitors with existing treatments, such as chemotherapy or immunotherapy, could further improve treatment outcomes. Incorporating pharmacogenomic profiling and ncRNA expression signatures in these studies would allow correlation between patient genotype, ncRNA regulation, and drug response, strengthening translational relevance. Clinical trials are also necessary to assess the safety, pharmacokinetics, and long-term efficacy of these compounds in cancer patients.

## 5 Conclusion

In conclusion, our study demonstrates the potential of *P. dulce* phytochemicals, specifically oleanolic acid and rutin, as isoform-selective AKT inhibitors. These compounds exhibit distinct isoform-specific binding profiles, with oleanolic acid targeting AKT1 and rutin targeting AKT2, making them promising candidates for targeted cancer therapy. By integrating pharmacogenomic and ncRNA perspectives, this study highlights a precision-medicine framework for exploiting isoform-specific vulnerabilities in the PI3K–AKT–mTOR pathway. This study provides a computational framework that may inform future efforts toward developing isoform-selective AKT modulators. However, the predicted interactions require extensive experimental validation before any therapeutic implications can be drawn.

## Data Availability

The datasets analyzed in this study are publicly available. Gene expression and genomic alteration data were obtained from The Cancer Genome Atlas (TCGA) through the GEPIA2 (http://gepia2.cancer-pku.cn/), TNMplot (https://tnmplot.com/analysis/), and cBioPortal (https://www.cbioportal.org) platforms. Survival analyses were performed using the Kaplan–Meier Plotter (http://kmplot.com/analysis). Protein structures were retrieved from the RCSB Protein Data Bank under accession codes PDB: 3QKL (AKT1) and PDB: 2JDO (AKT2). All in silico datasets generated in this study, including molecular docking, molecular dynamics, MM-GBSA, ADME, and DFT analyses, are available in Zenodo at https://doi.org/10.5281/zenodo.18324739.
